# Efficient TD3 based path planning of mobile robot in dynamic environments using prioritized experience replay and LSTM

**DOI:** 10.1038/s41598-025-02244-z

**Published:** 2025-05-26

**Authors:** Yunhan Lin, Zhijie Zhang, Yijian Tan, Hao Fu, Huasong Min

**Affiliations:** 1 School of Computer Science and Technology, Wuhan University of Science and Technology, Wuhan, 430081 China; 2 Hubei Province Key Laboratory of Intelligent Information Processing and Real-time Industrial System, Wuhan University of Science and Technology, Wuhan, 430081 China; 3 Institute of Robotics and Intelligent Systems, Wuhan University of Science and Technology, Wuhan, 430081 China

**Keywords:** Path planning in dynamic environment, Twin delayed deep deterministic policy gradient (TD3) algorithm, Reinforcement learning, Prioritized experience replay (PER), Long short-term memory (LSTM ), Engineering, Computer science

## Abstract

To address the challenges of sample utilization efficiency and managing temporal dependencies, this paper proposes an efficient path planning method for mobile robot in dynamic environments based on an improved twin delayed deep deterministic policy gradient (TD3) algorithm. The proposed method, named PL-TD3, integrates prioritized experience replay (PER) and long short-term memory (LSTM) neural networks, which enhance both sample efficiency and the ability to handle time-series data. To verify the effectiveness of the proposed method, simulation and practical experiments were designed and conducted. In the simulation experiments, both static and dynamic obstacles were included in the test environment, along with experiments to assess generalization capabilities. The algorithm demonstrated superior performance in terms of both execution time and path efficiency. The practical experiments, based on the assumptions from the simulation tests, further confirmed that PL-TD3 has improved the effectiveness and robustness of path planning for mobile robot in dynamic environments.

## Introduction

Mobile robot is a type of robotic system that can sense its environment and its own state through sensors, and achieve target-directed autonomous movement in environments with obstacles. Path planning is a key area of research in mobile robotics. Its primary task is to find an optimal, collision-free path from start to a target in an environment with obstacles. This path must satisfy several criteria: it should be as smooth, short and time-efficient as possible.

Path planning integrates local and global state information in robotic systems. In different scenarios, the system generates optimal or suboptimal path planning decisions. Standard path planning is categorized into traditional algorithms and machine learning based algorithms^1^. Traditional algorithms include global planning and local planning strategies.

The global planner generates the optimal path for a robot from start to target based on a prior map. The local path planner is responsible for adjusting the path in real time as the robot navigates, based on the information it perceives about the external environment to respond to obstacles. Traditional path planning algorithms include Simulated Annealing (SA), Artificial Potential Field (APF), Rapidly-exploring Random Trees (RRT), and Probabilistic Roadmap. The APF method, proposed by Khatib^2^, was designed to address obstacle avoidance problems. It works by generating an attractive force toward the target and a repulsive force away from obstacles. Path planning is achieved by calculating the resultant force at various points. Our previous work in^3^, based on the APF method, combined the case-based reasoning approach with the APF method to achieve real-time dynamic obstacle avoidance and improve path planning performance. Belanov et al.^4^ implemented the D*Lite method, which uses a heuristic reverse search mechanism, for path planning in unknown environments with variable start positions and a fixed target position. Akishita et al.^5^ conducted a study on obstacle avoidance for autonomous mobile robot using Laplacian methods. Seder et al.^6^ combined the focused D* search algorithm with the dynamic window local obstacle avoidance algorithm and used a jump point search method to obtain global information for path planning, thereby achieving path planning. The path planning problem can be solved using graph search methods such as depth-first search, breadth-first search, and Dijkstra’s algorithm. Alternatively, heuristic search methods can be used, including the A*^7^ algorithm and the D*^8^ algorithm. However, traditional path planning algorithms rely on precise maps, and in path planning algorithms, global path planning and local path planning are independent of each other. They require separate configurations based on different scenarios. This makes it difficult for traditional path planning algorithms to adapt to complex, unstructured, and dynamic environments. The research of path planners without maps that exhibit a certain degree of generalization, robustness, and adaptability is of great importance.

Considering these challenges, recent years have seen a shift towards machine learning-based path planning algorithms, which offer the potential to address these limitations and adapt to complex, unstructured, and dynamic environments. Machine learning-based path planning algorithms can be further categorized into supervised learning-based and reinforcement learning-based path planning algorithms^9^. In supervised learning, the agent plans effective paths by learning the mapping relationship between inputs and outputs. Motion Planning Networks (MPNet)^10^ is a general near-optimal heuristic approach that utilizes neural networks to learn path planning in both known and unknown environments. It receives environmental information, such as raw point clouds generated by depth sensors, as well as the robot’s start and target positions, and recursively invokes itself to generate bi-directional connectable paths. In^11^, Khan et al. investigated the feasibility of using Graph Neural Networks (GNNs) to solve conventional motion planning problems. However, supervised learning-based path planning algorithms rely on labeled data and have limited generalization capabilities, posing challenges when dealing with complex and dynamic environments.

With the development of reinforcement learning (RL), RL-based path planning methods have gradually become a new research focus^12^, as they can operate independently of prior map data during the training phase and enhance generalization capabilities by interacting with different scenarios. In unknown environments, RL can continuously learn and experiment to plan effective paths^13,14^. To address the computational challenges of continuous state spaces, neural networks were introduced into RL, leading to the development of deep reinforcement learning (DRL)^15,16^. DRL combines RL’s decision-making ability with deep learning’s perception capabilities, bridging the gap between high-dimensional inputs and actions, and has thus been widely applied in mobile robot path planning. Xin et al.^17^ were the first to apply Deep Q-Networks (DQN) to the path planning problem of mobile robot, making significant progress in discrete action control problems. Escobar-Naranjo et al.^18^ introduces a novel algorithm that integrates reinforcement learning with DQN to empower an agent with the ability to execute actions, gather information from a simulated environment in Gazebo, and maximize rewards. In^19^, Schaul et al. proposed a prioritized experience replay (PER) strategy, which improved the experience replay method of the DQN algorithm by assigning weights to experiences and prioritizing the replay of experiences with high weights to speed up the convergence of the model. However, the DQN-based algorithm is designed to solve path planning problems in discrete spaces. If used in continuous spaces, additional discretization is required. Even with this discretization, the actions may lack precision, which could potentially limit the quality of path planning. In^20^, Lillicrap et al. proposed DDPG (Deep Deterministic Policy Gradient), an extension of DQN that introduces a policy (Actor) and value function (Critic) architecture to support deterministic learning of continuous actions. However, the DDPG algorithm suffers from a high proportion of illegal policies due to the lack of policy action filtering, resulting in low training efficiency and slow convergence speed. Dong et al,^21^ accelerated deep reinforcement learning training by using a small amount of prior knowledge to reduce trial-and-error iterations. They employed an adaptive exploration method using the $\varepsilon$-greedy algorithm to speed up convergence. However, the overestimation issue may arise in practical applications of the DDPG algorithm, potentially affecting the accuracy and efficiency of policy learning. Scott Fujimoto et al.^22^ introduced twin delayed deep deterministic policy gradient (TD3). By incorporating dual Q-values and delayed update strategies, TD3 addresses the problem of network overestimation observed in DDPG. Several improvements have been made to the TD3 algorithm in previous studies. For instance, TD3-KDHER^23^ proposed an innovative approach by incorporating Kernel Density Estimation (KDE) to effectively utilize data in the experience replay buffer to fit a probability density model, thereby optimizing the selection strategy for pending experiences and new targets. Additionally, In^24^, improved TD3 was combined with RRT* to reduce path tortuosity and speed up convergence, yielding smoother and more efficient trajectories. Other studies^25^ have applied the TD3 algorithm to multi-robot path planning problems, demonstrating its flexibility and efficiency in complex environments. In^26^, proposed an innovative method that combines the APF with the TD3 algorithm, introducing the APF-TD3 framework. This approach effectively addresses the path planning challenges in automated terminals. The method not only ensures path smoothness but also improves the safety and feasibility of the path through policy optimization using reinforcement learning. Similar to our approach,^27^ adds PER and designs a dynamic delay update strategy, which can reduce the impact of value estimation errors, thereby improving the success rate of path planning and reducing training time. However, these methods have not to address the path planning challenges for mobile robots in dynamic environments.

In summary, traditional path planning algorithms rely on known map information, while reinforcement learning-based algorithms exhibit slow convergence speeds and insufficient perception of dynamic obstacles^28,29^. In the field of reinforcement learning algorithms, TD3 stands out as a powerful approach. However, in complex dynamic environments, the TD3 algorithm still faces (a) improvements in sample utilization efficiency and (b) the use of feedforward neural networks, which may not be effective enough in handling environments with temporal dependencies.

To address the issues encountered by TD3, we propose PL-TD3, a path planning algorithm that enhances the TD3 algorithm by incorporating PER and LSTM. The main contributions of this paper are summarized as follows:

1. To address the challenges of low sample utilization and slow convergence, we use the PER technique to enhance sample efficiency. The PER technique assigns higher weights to high-value samples, allowing the agent to learn effective strategies more quickly during training. This improves the efficiency and accuracy of selecting and updating high-value samples.
2. To address the issue of time-dependent problems that cannot be resolved by feedforward neural networks in the TD3 algorithm, LSTM networks were introduced to capture temporal dependencies. This helps the agent consider past states and actions when making decisions. The recurrent connections leverage the history of actions and states, facilitating more effective decision-making and enhancing path planning.
3. Compared to our previous conference paper^30^, which initially verified PL-TD3’s efficiency in simulations. In this paper, we conducted a more systematic theoretical analysis and comprehensive experimental validation of the effectiveness of PL-TD3 in both simulation and real robot scenarios.

The overall structure of the paper is as follows: Section 2 introduces the PL-TD3 algorithm, followed by Section 3 which validates the algorithm through simulation experiments. In Section 4, real experiment evidence is presented to demonstrate the effectiveness of the PL-TD3. Section 5 discusses the advantages and disadvantages of the paper, and finally Section 6 summarizes the findings and concludes the paper.

## Proposed PL-TD3

### The structure of PL-TD3

TD3 is a reinforcement learning algorithm applied in continuous action spaces. It incorporates critical improvements on the foundation of DDPG, addressing several issues common in DDPG, such as overestimation and instability during the training process. When applied to path planning in dynamic environments, the TD3 algorithm may still face challenges related to sample utilization efficiency and handling temporal dependencies effectively. These obstacles can impact the algorithm’s performance and effectiveness in navigating complex and dynamic environments.

**Fig. 1:**
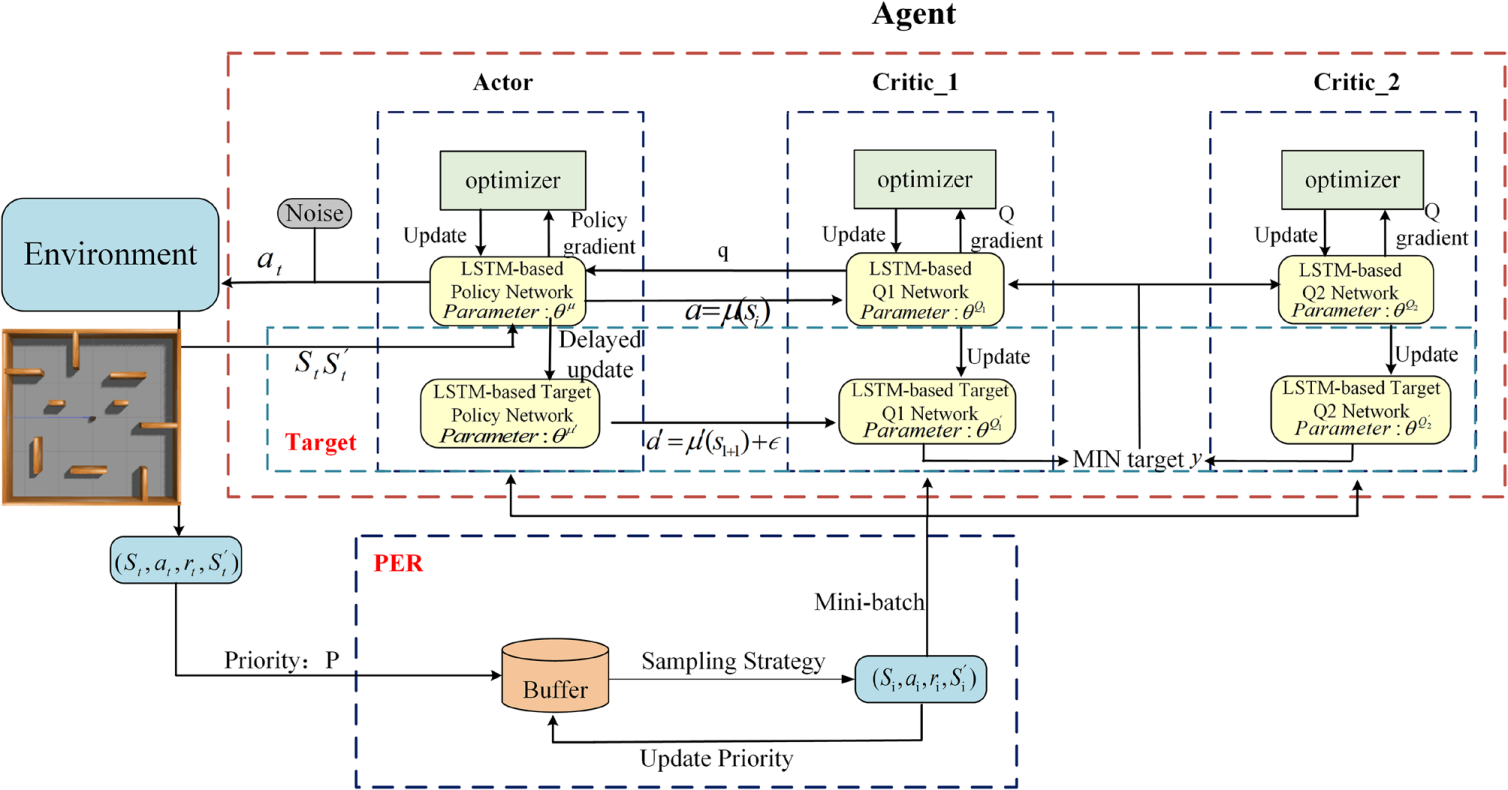
The structure of PL-TD3 algorithm.

In this paper, by integrating PER and LSTM into the TD3 algorithm, the sampling efficiency of TD3 can be increased and its performance in environments requiring long-term planning can be improved. These enhancements make TD3 more effective and robust in a wider range of application scenarios. The overall structure of the algorithm is shown in Fig. 1.

### Long short-term memory

**Fig. 2:**
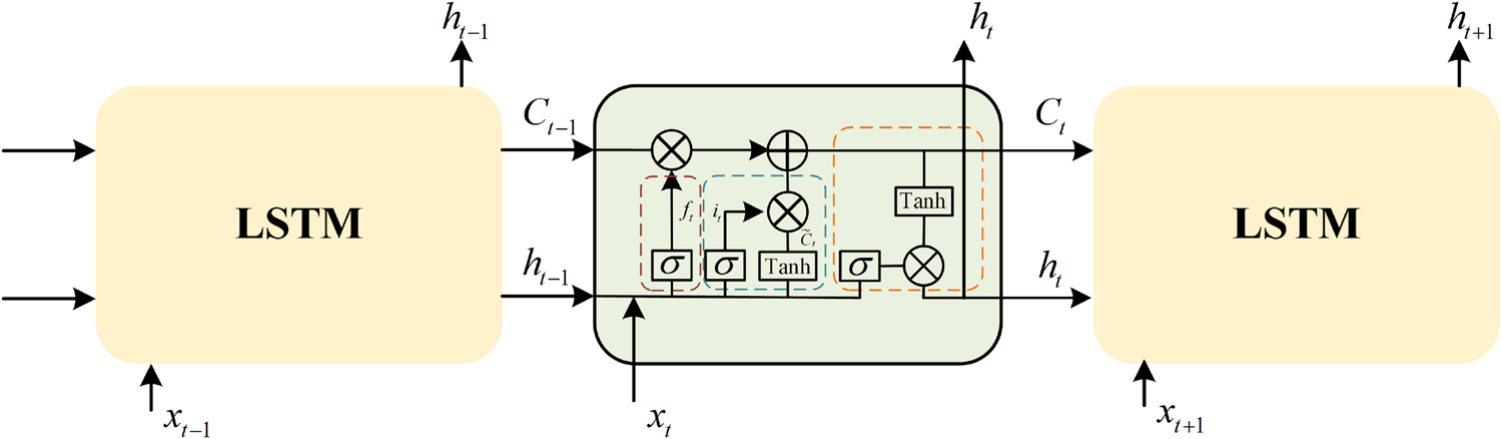
Structure diagram of LSTM neural networks.

In terms of handling temporal dependencies effectively. TD3 typically uses feedforward neural networks to handle environments with temporal dependencies. However, when dealing with environments with temporal dependencies, feedforward neural networks are not effective enough. LSTM is a special type of Recurrent Neural Network (RNN) that is well suited for processing data with temporal sequence dependencies. However, traditional RNNs are prone to issues such as gradient explosion. The LSTM network effectively addresses the problems of vanishing and exploding gradients encountered by traditional RNNs in handling long-term dependencies by introducing three key gates: the forget gate (f), the input gate (i), and the output gate (o). The forget gate is responsible for determining which information to discard, the input gate decides what information to update, and the output gate controls the information passed to the next hidden state. The collaborative operation of these three gates enables LSTMs to selectively retain and transmit important information, thereby exhibiting excellent performance in processing time series data. A structure diagram of LSTM neural networks is shown in Fig. 2.

**Fig. 3:**
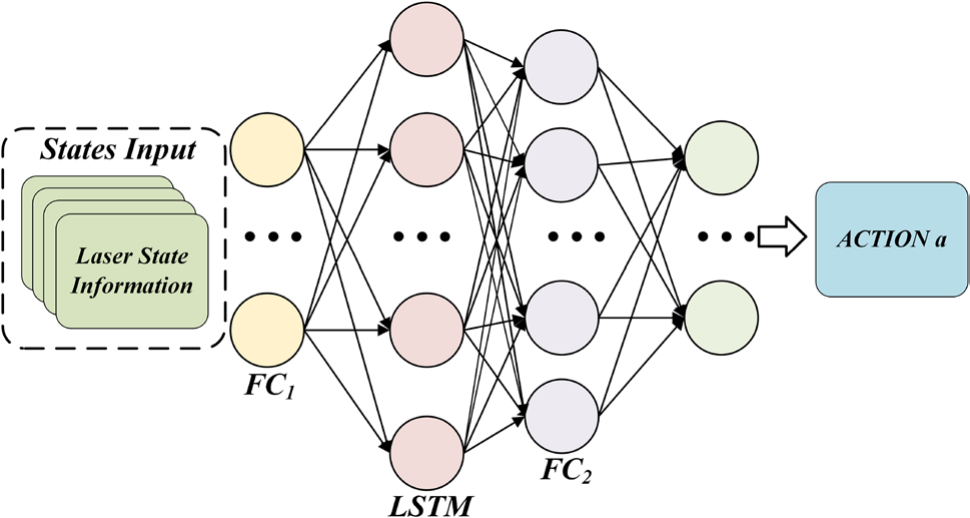
Structure diagram of LSTM+TD3 network.

In the framework, both the Actor and Critic components consist of an LSTM structure followed by two fully connected layers. Figure 3 illustrates the network structure of the TD3 algorithm after incorporating LSTM. This diagram represents the final network architecture of the LSTM-TD3 algorithm. The actor network initiates with a fully connected layer, denoted as $FC_{l}$, which takes the state feature vector s as input and employs the ReLU activation function to facilitate enhanced feature extraction. Subsequently, a memory-capable LSTM layer is integrated, enabling the algorithm to merge historical and current states for more informed decision-making. Following the LSTM layer, a fully connected layer $FC_{2}$, processes the LSTM layer’s output, further refining the LSTM’s features. The sigmoid activation function is utilized to ensure that the resulting action a falls within the desired range. In the TD3 algorithm, the Critic network receives the state vector s and the action vector a as inputs. Two independent Critic networks are employed to mitigate overestimation issues in Q-value estimation, ultimately producing the Q-value used to update the Actor network.

### Twin delayed deep deterministic policy gradient

The data input to the experience pool, labeled $s_i,a_i,r_i,s_{i+1}$ represent the current state of the robot, the action, the reward received, and the new state after performing the action, respectively. The $s_i$ is composed of the data from the LiDAR, primarily including the distance between the robot’s current position and the target, the distance between the robot and the nearest obstacle, and the angle between the robot’s immediate front and the nearest obstacle. The $a_i$ is the predicted value of the action by the actor policy network based on the current $s_i$ of the robot. The $s_i$, with a frequency of 20 Hz, controls the angular and linear velocity of the robot. The $s_{i+1}$ represents the new state the robot reaches after executing the $a_i$. The $r_i$ is received by the robot after executing the action $a_i$.

During the iterative process, when the number of experiences in the experience pool exceeds *N*, the algorithm systematically uses the PER strategy to select a batch of experiences from the pool. It then calculates the Temporal Difference error (TD error) for each selected experience, where TD error represents the error for each experience. Based on the size of the TD error, the algorithm updates the priorities of these experiences in the experience pool. These experiences are subsequently used to update both the actor network and the critic network. The updating procedure for the Critic network is defined by Eqs. (1)–(4)^31^.

1 $$\begin{aligned} & a^{'} = \mu _{\theta ^{\mu }}^{'}(s_{i+1}^{'}) + \varepsilon \end{aligned}$$

2 $$\begin{aligned} & y=r+\gamma \min \{Q_{\theta ^{Q_1^{\prime }}}(s_{i+1},a^{\prime }),Q_{\theta ^{Q_2^{\prime }}}(s_{i+1},a^{\prime })\} \end{aligned}$$

3 $$\begin{aligned} & \theta ^{Q_1} \leftarrow \arg \min _{\theta ^{Q_1}} N^{-1}\sum (y - Q_{\theta ^{Q_1}}(s_i, a_i))^2 \end{aligned}$$

4 $$\begin{aligned} & \theta ^{Q_2} \leftarrow \arg \min _{\theta ^{Q_2}} N^{-1} \sum _{i} (y - Q_{\theta ^{Q_2}}(s_i, a_i))^2 \end{aligned}$$

Herein, $\theta ^ {Q}$ represents the parameters of the critic network, while $\theta ^{\mu }$ indicates the parameters of the actor network. $\gamma$ represents the discount rate, and $\epsilon$ is the Gaussian noise added to the action space. The Actor network uses a delayed update approach, which means that for every three updates of the Critic network, the Actor network is updated once. As the Actor network is updated by maximizing the cumulative expected return, it relies on the Critic network for evaluation. If the Critic network is unstable, the Actor network will naturally oscillate. Therefore, it is plausible to increase the update frequency of the critic network above that of the actor network, waiting for the critic network to stabilize before updating the actor network. This serves to mitigate problems such as overestimation. Its update method is given by Eq. (5).

5 $$\begin{aligned} \nabla _{\theta ^{\mu }}J(\theta ^{\mu })=N^{-1}\sum \nabla _aQ_{\theta ^{q_1}}(s_i,a)\Big |_{a=\mu _{\theta ^{\mu }}(s_i)}\nabla _{\theta ^{\mu }}\mu _{\theta ^{\mu }}(s_i) \end{aligned}$$

Whenever the actor network is updated, all target networks are updated simultaneously using a soft-update strategy, as illustrated by the update methods described in Eqs. (6) and (7).

6 $$\begin{aligned} & \theta ^{Q'_j} \leftarrow \tau \theta ^{Q_j} + (1-\tau ) \theta ^{Q'_j} \end{aligned}$$

7 $$\begin{aligned} & \theta ^{\mu '} \leftarrow \tau \theta ^{\mu } + (1-\tau ) \theta ^{\mu '} \end{aligned}$$

### Experience replay strategy

In terms of sample efficiency in reinforcement learning, agents update their parameters incrementally upon receiving experiences and discard those experiences after they have been used for training. This can lead to algorithmic instability or divergence, as well as discarding potentially valuable experiences for future use.

PER is a reinforcement learning technique that addresses the above issues. At first, the agent learns to make decisions by interacting with the environment and receiving reward feedback. The introduction of a replay buffer creates an experience replay pool that stores previously observed transitions by the agent, including states, actions, rewards and subsequent states. During training, the agent randomly samples experience batches from this replay buffer to update its neural network parameters. Through experience replay, we can break the correlation between samples, reducing the risk of overfitting, and reuse samples from the buffer, increasing sampling efficiency. In TD3, the default experience replay mechanism treats all experiences equally, which can lead to suboptimal learning outcomes, as some experiences may be more informative or critical to the learning process than others. That is why we introduce PER. It selects important experiences for learning. Similarly, the agent performs actions in the environment, collecting transitions that are stored in the experience replay pool. Additionally, each new experience is assigned an initial priority value. We measure the priority of each experience by calculating the TD error. A larger TD error value indicates a greater potential for improvement, making the experience more significant and therefore a higher priority.

The experience replay mechanism in PL-TD3, based on the TD3 framework, uses weight-based prioritized experience replay. The PER mechanism determines the importance of experiences based on the TD error of the experiences. The TD error is the difference between the estimated action value and the current output of the value function. A higher TD error indicates greater inaccuracy in the output of the current value function, which means greater usability of the corresponding experience. The calculation of the TD error, denoted as $\delta _{i}$ is defined by Eq. (8).

8 $$\begin{aligned} \delta _i = r_i + \gamma \min \{Q_{\theta ^{Q1'}}(s_{i+1}, \mu '_{\theta ^{\mu '}}(s_{i+1})), Q_{\theta ^{Q2'}}(s_{i+1}, \mu '_{\theta ^{\mu '}}(s_{i+1}))\} - Q_{\theta ^{Q1}}(s_i, a_i) \end{aligned}$$

$s_{i}$ represents the state of the robot at time *i*, $a_i$ denotes the action performed by the robot in state $s_i$ , $s_{i+1}$ is the new state the robot reaches after performing the action, $r_i$ is the reward received by the robot for taking the action, and $\gamma$ is the discount factor.

The probability of each experience being replayed is calculated as shown in Eq. (9).

9 $$\begin{aligned} P(i)=\frac{p_i^\alpha }{\sum _kp_k^\alpha } \end{aligned}$$

TD error is the Temporal Difference error for each experience, and $\alpha$ is a parameter used to adjust the significance of the TD error. When $\alpha$ equals 1, the TD error is utilized directly as its numerical value. If $\alpha$ is less than 1, it can diminish the impact of experiences with high TD errors and enhance the influence of those with low TD errors. There are two methods for defining TD errors: Proportional Priority $p_i=|\delta _i|+\varepsilon$ and Rank-based Priority $p_i=1/rank(i)$. This paper adopts the latter. 1/*rank*(*i*) is determined based on the ranking of $|\delta _i|$.

To ensure that experiences with unknown TD errors, such as new samples, are replayed at least once, they are prioritized to the front. Subsequently, during each replay, the sample with the maximum TD error is selected for playback. When the TD error of a particular experience is zero, its priority is set to the minimum priority level. This ensures that an experience’s priority is directly proportional to its TD error, while also ensuring that samples with the lowest priority have a non-zero probability of being selected.

### Design of the reward function

The reward function is a benchmark for evaluating robot actions in reinforcement learning algorithms. The design of the reward function in this paper is as follows: If the robot reaches the target area, it is given the maximum positive reward $R_{reach}$, If the robot collides with an obstacle, the maximum negative reward $R_{colide}$ is given. If the robot neither collides nor reaches the target area, its reward is composed of the yaw angle reward $R_{1}$ multiplied by the distance reward $R_{2}$ between the robot and the target point, plus the minimum distance reward $R_{3}$ between the robot and obstacles, If there is a significant difference between the robot’s yaw angle and the direction of the target location, the robot may move in the opposite direction even when it is close to the target location. Therefore, the product of $R_{1}$ and $R_{2}$ can determine both the sign and the amount of the reward based on the distance. The calculation of the reward value is shown in Eq. (10).

10 $$\begin{aligned} Reward={\left\{ \begin{array}{ll}R_{reach},\text {Arrival at destination}\\ R_{colide},\text {Collision with obstacles}\\ R_1\times R_2+R_3,\text {Other positions}\end{array}\right. } \end{aligned}$$

Calculating the robot’s yaw angle reward $R_{1}$ is shown in Eq.(11).

11 $$\begin{aligned} R_1=4\times (1-abs(\alpha )) \end{aligned}$$

Where $\alpha$ represents the radian measure of the angle between the robot’s forward direction and the target, with a value range of $$\begin{bmatrix}-\pi ,\pi \end{bmatrix}$$.

The calculation of the distance reward $R_{2}$ between the robot and the target point is given by Eq. (12).

12 $$\begin{aligned} R_2=2^{\frac{d_{t-1}}{d_t}} \end{aligned}$$

Where $d_{t-1}$ and $d_{t}$ represent the distance between the robot and the target point at the last instance and the current distance between the robot and the target point, respectively.

The calculation of the minimum distance $R_{3}$ between the robot and obstacles is represented as shown in Eq. (13).

13 $$\begin{aligned} R_3={\left\{ \begin{array}{ll}-5,min_{obs}<0.5\\ 0,min_{obs}\ge 0.5\end{array}\right. } \end{aligned}$$

Where $min_{obs}$ is the shortest distance between the robot and the nearest obstacle at the current moment. When $min_{obs}$ is less than 10 cm, it is considered a collision. Therein, the robot is considered as a mass point, and the body of mobile robot is included in the calculation of $min_{obs}$. When the robot is close to an obstacle, it is prone to collisions and needs a negative reward to maintain a safe distance from the obstacle. However, if the safe distance maintained by the robot from the obstacle is too large, or the negative reward given is too large, it may affect the robot’s obstacle avoidance strategy and prevent it from navigating through narrow areas. In this paper, we adjust the threshold of $min_{obs}$ and the negative reward through experiments. It is found that the performance of the algorithm is optimal when the threshold of $min_{obs}$ is set to 0.5 and the negative reward is set to -5.

Based on the above structure, we summarize the process of the PL-TD3 algorithm as follows Algorithm 1

**Algorithm 1:**
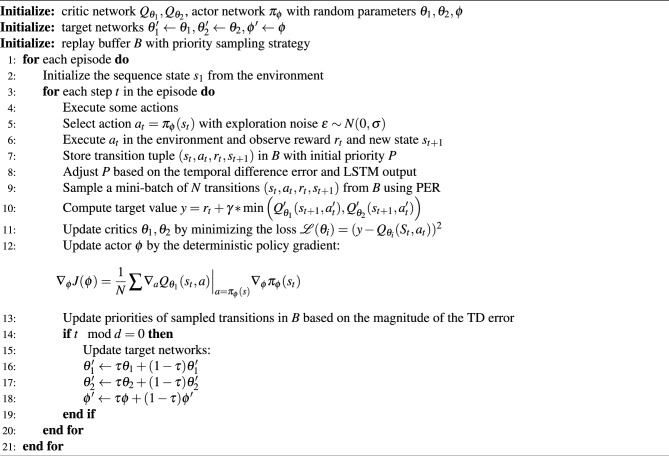
PL-TD3.

## Simulation experiment

### Experimental environment and parameter settings

To verify the effectiveness of PL-TD3, we built a 3D map simulation environment using ROS (Robot Operating System) under the hardware conditions of AMD R5 3600X, NVIDIA GTX1650, and 16G RAM, running on an Ubuntu 16.04 system. We designed experiments for static and dynamic obstacles testing, as well as generalized testing to perform comparative analyses of the methods before and after the improvement, each simulation was tested 100 times. The location of the robot and moving obstacles is obtained by reading the position messages published by ROS. The specific hyperparameter settings are shown in Table 1.

**Table 1:** Hyperparameter settings of experiment.

Parameter Name	Value
Discount Factor	0.98
Learning Rate	0.0008
Number of Hidden Units	256
Episode Number	1,500
Max Steps	500
Experience Replay Buffer	100,000
$$(R_{reach}, R_{collide})$$	(1000, -800)

The number of attempts is set to 1,500. The buffer size is set to 100,000, as recommended by reference^32^. This size is an empirical choice demonstrated in practice to balance efficiency, stability, and resource consumption effectively.

**Fig. 4:**
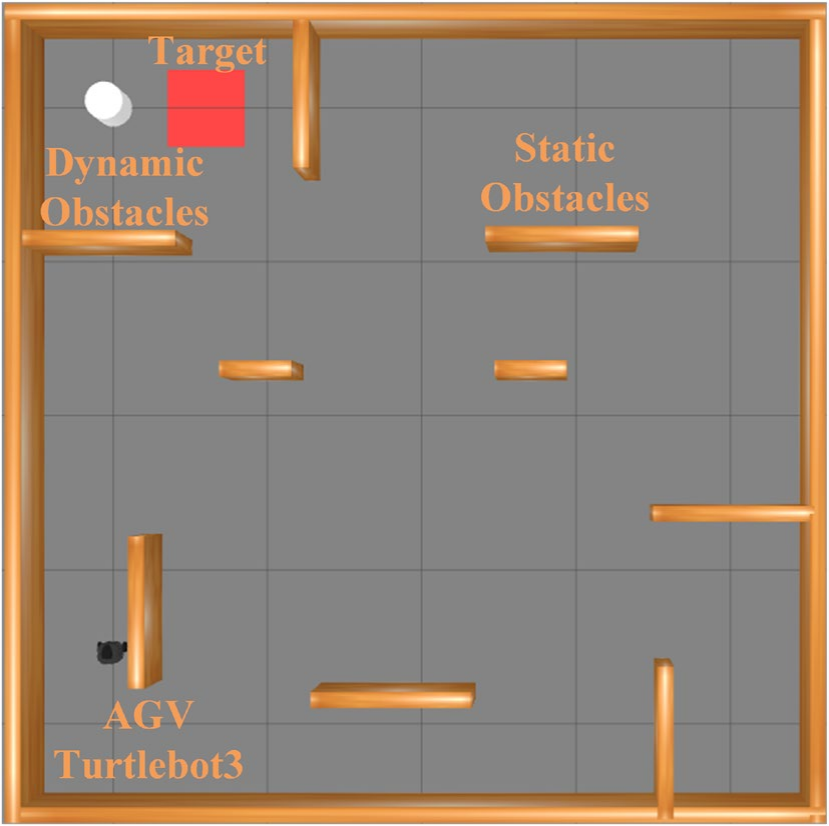
Diagram of training environment definition.

The training environment is shown in Fig. 4. The brown-yellow blocks represent static obstacles, while the white cylinders represent dynamic objects. The red squares indicate the target points. After completing 1,500 training epochs (each step takes 1 second, and each episode contains 500 steps, resulting in a maximum training time of 1500 episodes * 500 steps * 1 second, which is approximately 200 hours under ideal conditions without collisions. Based on our observations, the average training time is approximately 100 hours.), the resulting model was evaluated over 100 independent trials, and the average performance across these trials was adopted as the definitive evaluation metric. To enhance the mobile robot’s perception of the training environment, we have adopted a method where the initial starting point is fixed, while the target point is randomized during training. This approach differs from most studies, as it is designed to optimize the mobile robot’s ability to reach the target point in the fewest steps possible, maximizing rewards. Upon the mobile robot’s arrival at a target point, instead of terminating the current training episode, a new target point is randomly assigned. This allows the robot to reach as many targets as possible within the specified max steps 500 if the step limit is reached in an epoch, training proceeds to the next epoch, thereby generating smoother, shorter, and more efficient paths.

### Dynamic environment experiment

**Fig. 5:**
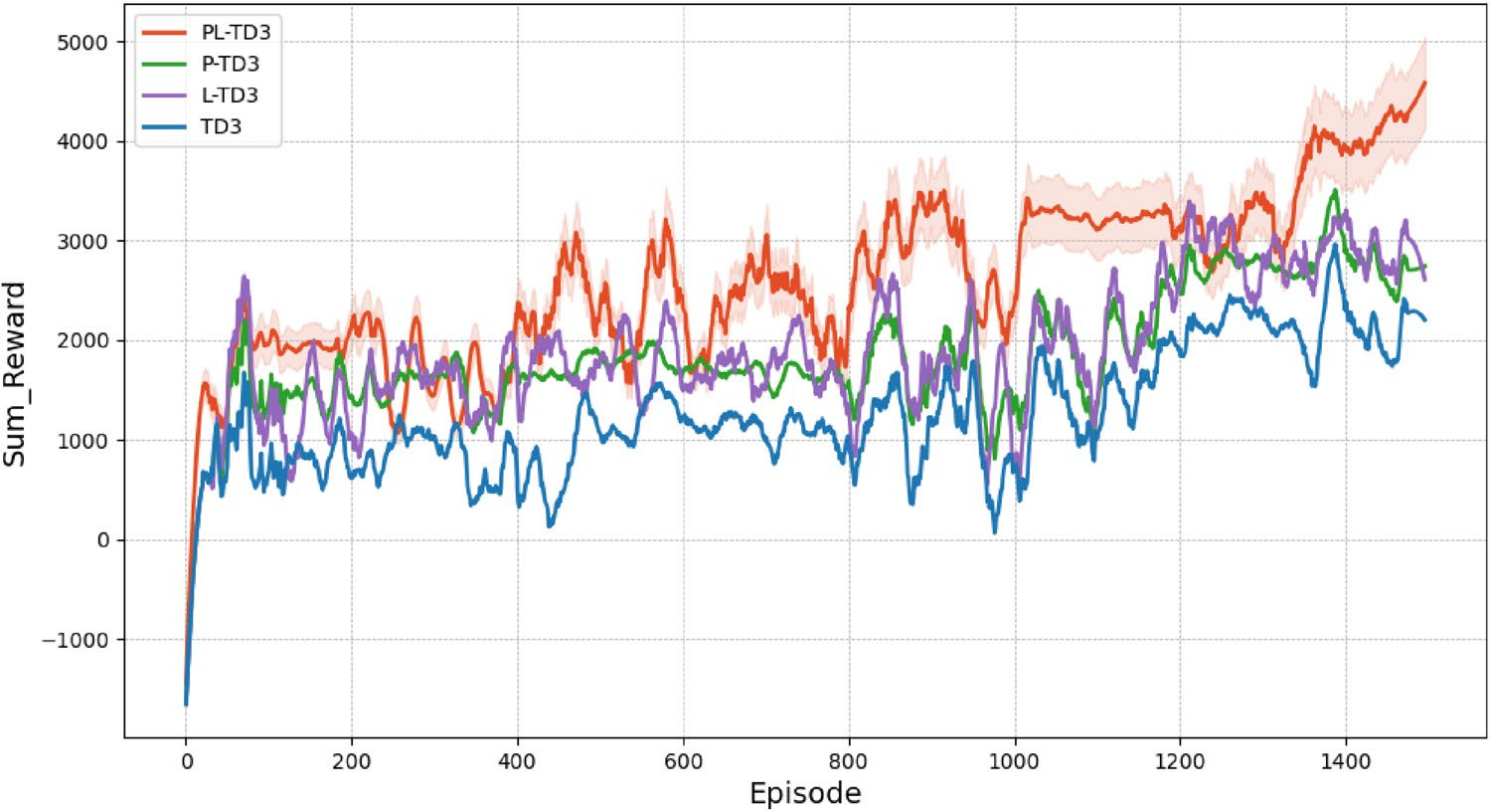
Comparison of training rewards of PL-TD3, P-TD3, L-TD3, and TD3 in the training scenario.

In this section, several reinforcement learning methods are tested in an environment with static and dynamic obstacles, including TD3, P-TD3 (PER+TD3), L-TD3 (LSTM+TD3), and PL-TD3 (the proposed method). Figure 5 shows the reward values for PL-TD3, P-TD3, L-TD3, and TD3 algorithms after 1500 training rounds. The experimental results demonstrate that PL-TD3 outperforms other algorithms in both convergence speed and maximum cumulative reward, indicating that the PER strategy significantly enhances the algorithm’s convergence speed. Additionally, by capturing temporal dependencies in time series, LSTM effectively retains and utilizes historical action-state information, enhancing the agent’s perception of the environment and enabling more precise decision-making. This allows the robot to avoid obstacles and reach the target position through shorter paths.

It is important to note that in our training environment, the target points are generated randomly, which results in varying distances and difficulties for the robot in reaching these points. This randomness in the target points not only enhances the generalization ability of our method but also naturally leads to fluctuations in the rewards collected by different algorithms during training. As shown in Fig. 5, our proposed method demonstrates significant advantages in both convergence speed and cumulative reward. On one hand, PL-TD3 requires approximately 400 episodes to reach a cumulative reward of 2000 (reaching the target points at least once), while other methods show more fluctuations, highlighting the significant improvement in convergence speed. On the other hand, considering the overall trend, after 1200 episodes, the cumulative reward ranking from high to low is: PL-TD3, L-TD3, P-TD3, and TD3, further proving the advantages of our method in optimizing decision-making and improving efficiency.

**Fig. 6:**
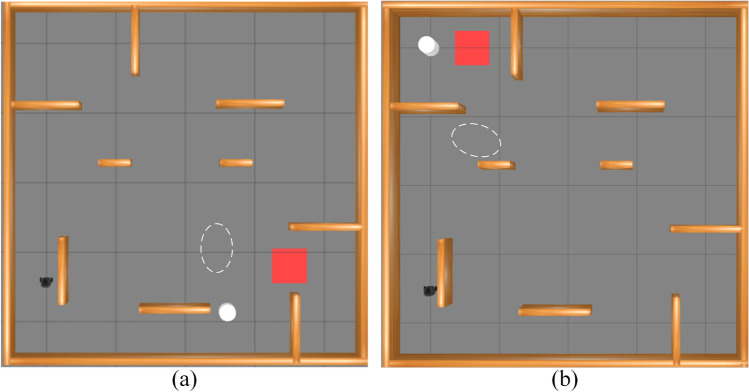
The simulation environment experimental scenarios: (**a**) the robot encounters the human in a wide area; (**b**) the robot encounters the human in a narrow area.

**Fig. 7:**
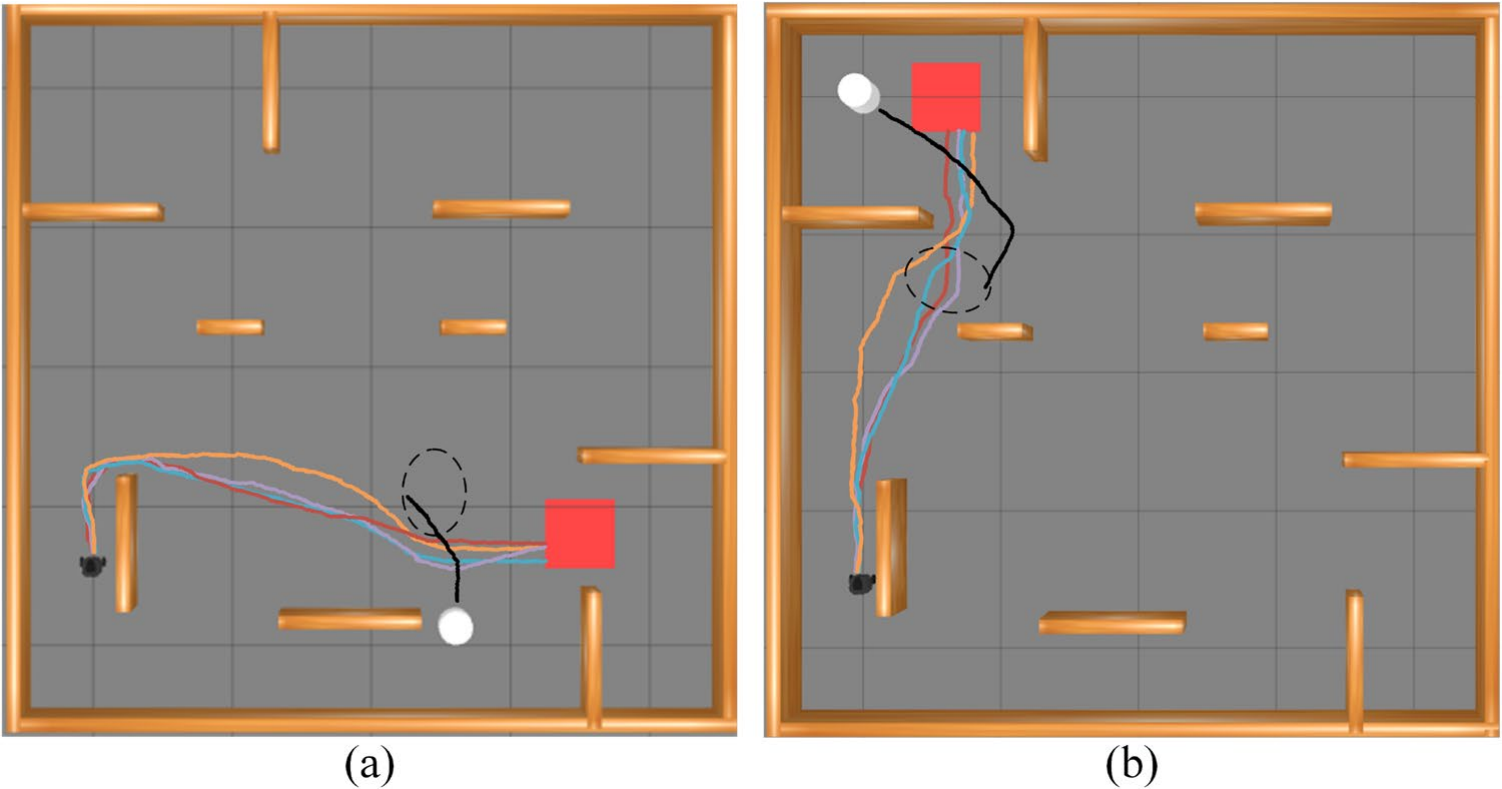
Examples of simulation results from different path planning methods in the training scenarios: (**a**) the robot encounters dynamic obstacles in a wide area; (**b**) the robot encounters dynamic obstacles in a narrow area. The trajectory generated by PL-TD3 is shown in red, L-TD3 in purple, P-TD3 in blue, and TD3 in orange, while the black paths indicate the motion of the dynamic obstacles.

Two experiments were conducted to evaluate obstacle avoidance in both wide and narrow area scenarios. Figure 6 illustrates these areas in the test environment. The robot, shown in black, departs from a designated start point and must reach the red rectangular target, while the white ellipse marks the region where it encounters pedestrians. Figure 7 shows the trajectories produced by the four algorithms: PL-TD3 (red), L-TD3 (purple), P-TD3 (blue), and TD3 (orange). Black paths trace the motion of the dynamic obstacles. PL-TD3 keeps closer to pedestrians and moves more directly toward the target, producing noticeably smoother paths. In contrast, the other algorithms, especially TD3, display weaker environmental awareness and often take longer detours, which greatly increase both travel distance and execution time. The detailed metrics are shown in Table 2. In the wide area scenario, PL-TD3 shortens the path by 1.27m, cuts execution time by 31s, and raises the success rate by 14 percentage points. In the narrow area scenario, it further reduces the path by 0.39m, saves another 10s, and boosts the success rate by 15 percentage points, confirming its superiority in dynamic obstacle avoidance and overall efficiency.

**Table 2:** Comparison of main evaluation indices for narrow and wide area scenarios in the simulation environment.

ENV	Algorithm Name	Main Evaluation Index		
		Average Path Length (m)$\downarrow$	Execution Time(s)$\downarrow$	Success Rate (%)$\uparrow$
ENV (Encounter in Wide Area)	PL-TD3	**5.27**	**67**	**87%**
	L-TD3	5.64	73	83%
	P-TD3	5.71	80	79%
	TD3	6.54	98	73%
ENV (Encounter in Narrow Area)	PL-TD3	**5.84**	**75**	**85%**
	L-TD3	5.93	81	78%
	P-TD3	5.86	77	77%
	TD3	6.23	85	70%

The bold type represents the algorithm having the best performance.

### Generalization testing experiment

**Fig. 8:**
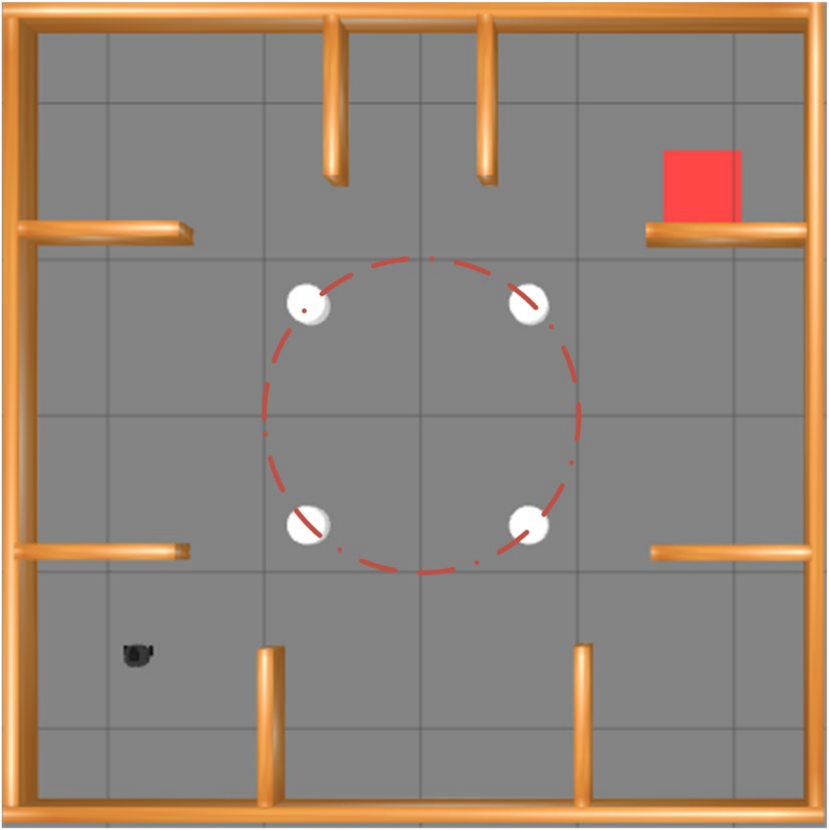
Generalization experiments. The red circles on the map represent obstacle trajectories, the robot’s current position is the starting point, and the red rectangle marks the target location.

The algorithms are evaluated for generalization in the simulated environment shown in Fig. 8. The robot starts in the bottom-left corner and must pass through an area marked by four white cylinders that represent pedestrians. Its target is to reach the red target region in either the top-left or top-right corner. The red circles indicate the simulated trajectories of pedestrian movement.

**Fig. 9:**
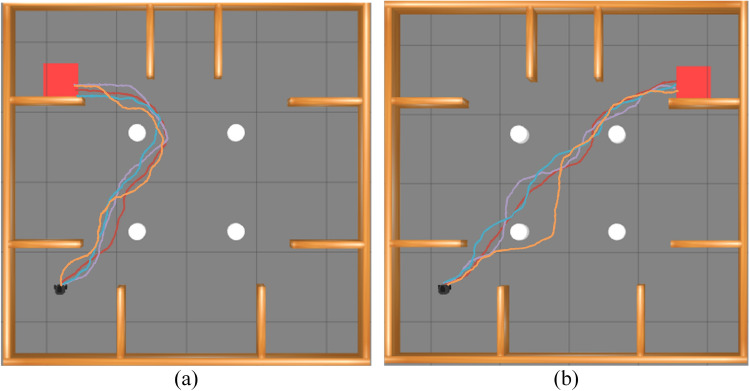
Examples of simulation results from different path planning methods in the testing experiments: (**a**) target position in the top-left corner; (**b**) target position in the top-right corner. The trajectory generated by PL-TD3 is shown in red, L-TD3 in purple, P-TD3 in blue, and TD3 in orange.

Table 3 summarizes the average path length, execution time, and success rate of each algorithm. The corresponding trajectories are shown in Fig. 9 (PL-TD3 in red, L-TD3 in purple, P-TD3 in blue, and TD3 in orange). The results indicate that PL-TD3 outperforms all other methods in success rate, path length, and execution time. In complex scenarios with multiple moving obstacles, A detailed comparison between the L-TD3 and P-TD3 algorithms shows that, in complex scenarios with multiple moving obstacles, L-TD3 shortens the average path length by 6.2% and reduces the execution time by 10.6% relative to P-TD3. These results demonstrate that incorporating an LSTM network-which leverages historical states and actions to capture temporal dependencies-markedly improves path-planning efficiency and overall performance in dynamic environments.

**Table 3:** Comparison of the main evaluation indices in the generalization experiments.

ENV	Algorithm Name	Main Evaluation Index		
		Average Path Length (m) $\downarrow$	Execution Time(s)$\downarrow$	Success Rate (%)$\uparrow$
ENV (Target on Top-Left Corner)	PL-TD3	**6.59**	**80**	**81%**
	L-TD3	6.73	87	79%
	P-TD3	6.86	87	76%
	TD3	7.12	98	65%
ENV (Target on Top-Right Corner)	PL-TD3	**6.84**	**85**	**81%**
	L-TD3	7.13	91	78%
	P-TD3	7.91	112	75%
	TD3	8.23	135	63%

The bold type represents the algorithm having the best performance.

## Practical robot experiment

**Fig. 10:**
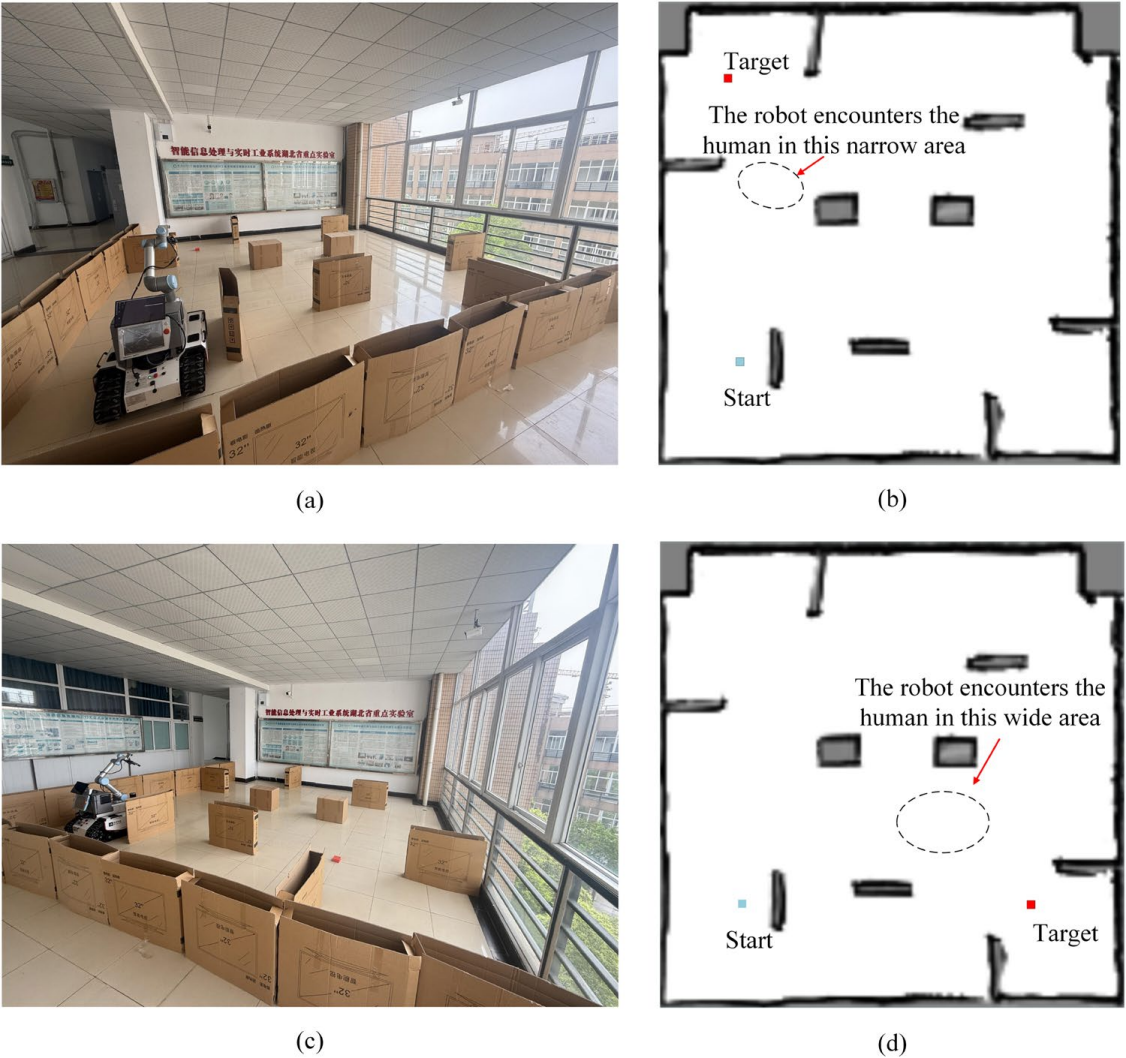
The real-world experimental environment: (**a**) The actual scene of the robot encountering the human in a narrow area; (**b**) The experimental setup of the robot encountering the human in a narrow area; (**c**) The actual scene of the robot encountering the human in a wide area; (**d**) The experimental setup of the robot encountering the human in a wide area.

To further verify the practicality of the PL-TD3 algorithm in real-world scenarios, we loaded a model trained for 1,500 episodes and tested it in a real environment. We designed an experimental environment comprising two scenarios, as shown in Fig. 10: the first involves robot-human encounters in narrow areas (Fig. 10a and b), and the second involves robot-human encounters in wide areas (Fig. 10c and d).

The real-world experimental setup is shown in Fig. 10a and c, with the test area measuring 8m $\times$ 8m. In Fig. 10b and d, the start point is marked with a blue dot and the target point with a red dot; cardboard boxes serve as static obstacles, and black dashed lines indicate the human-robot encounter zones. We selected the BUNKER AGV platform, produced by AgileX, for testing. The platform features a tracked chassis with independent suspension and built-in buffers, with overall dimensions of 1023mm$\times$778mm$\times$400mm. For high-precision navigation and obstacle avoidance, the robot is equipped with a Robosense RS-Helios-16P LiDAR featuring 16 laser beams.

### The parameter settings and evaluation metrics

The parameter settings for the PL-TD3 algorithm are as follows: a reward of 1000 ($R_{reach}=1000$) is assigned for the robot reaching the target position; a reward of -800 ($R_{colide}=-800$), is set for the robot colliding with an obstacle; the range of the robot’s angular velocity is set from -1 to 1 rad/s; and the range of the robot’s linear velocity is set from 0 to 0.8 m/s.

In this section, we use the success rate *C* of the robot reaching the target position as the evaluation metric, which is calculated using the formula is presented in Eq. 14

14 $$\begin{aligned} C=\frac{N_c}{N} \end{aligned}$$

Herein, $N_c$ is the number of times the robot successfully reaches the target position, and *N* is the total number of trials.

In this experiment, we compare the proposed PL-TD3, the baseline TD3, the global-search-based A* algorithm, and the sampling-based RRT algorithm. The average path length, the number of successful instances, the collision rate, and the success rate were selected as the primary evaluation metrics.

### Construction of the actual scenario map

**Fig. 11:**
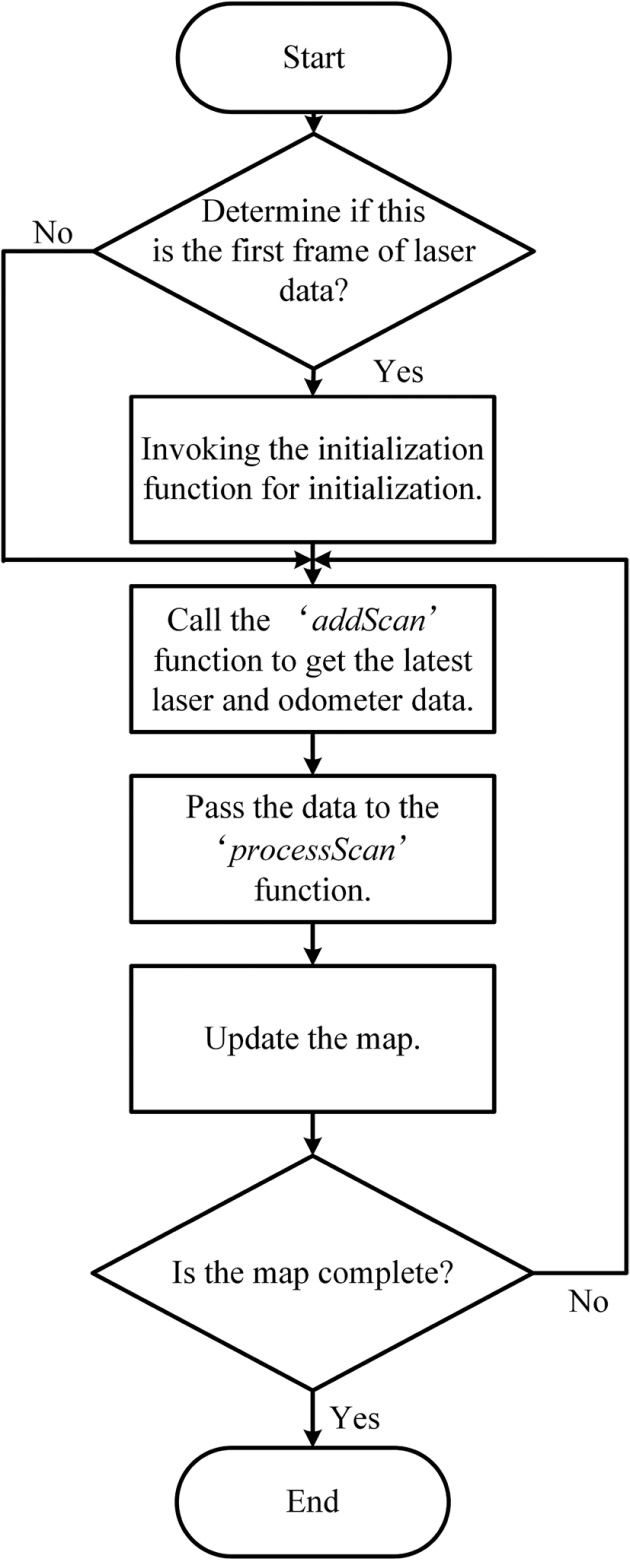
The gmapping process.

This section describes the construction of a two-dimensional grid map in the real experimental scene to support the path planning of our robot. The Gmapping^33^ method is used to construct the SLAM (Simultaneous Localization and Mapping) map. Gmapping is a SLAM algorithm based on the RBPF (Rao-Blackwellized Particle Filter), which estimates the robot’s pose using LiDAR and odometry data, and constructs the environmental map accordingly. The principle of map construction by the Gmapping algorithm is shown in Fig. 11.

**Fig. 12:**
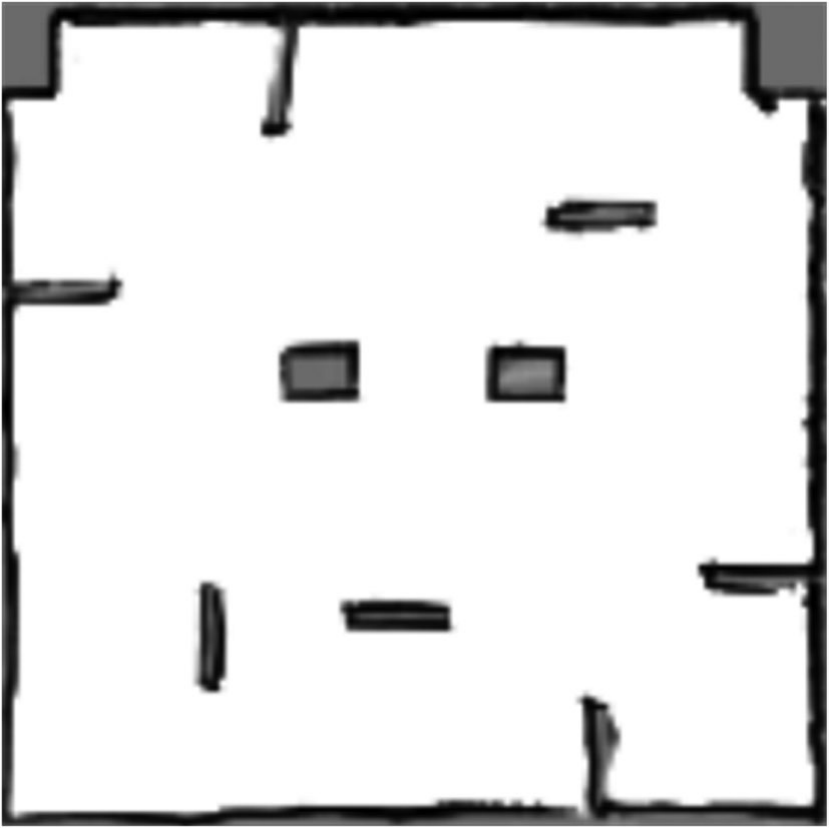
The PGM map of the actual scene..

In this algorithm, the ’addScan’ function is used to acquire the robot’s odometry pose corresponding to the laser timestamp, while the ’processScan’ function assists in robot localization using laser data, thereby enabling the acquisition of more precise robot position information. Once these accurate coordinates are obtained, the map can be updated based on the robot’s location and the LiDAR data. The SLAM map of the actual scene is shown in Fig. 12.

### The results of path planning

In this section, the PL-TD3 algorithm was tested 100 times. Based on the pre-obtained 2D grid map, the robot’s position is determined and updated during the online stage of the SLAM algorithm. The PL-TD3, TD3, RRT, and A* algorithms were compared. Through experimental verification, it has been confirmed that the A* and RRT algorithm does not have dynamic obstacle avoidance capability. This is because the A* and RRT algorithm is a static path planning algorithm that assumes a fixed obstacle configuration during planning and lacks mechanisms for real-time detection of dynamic obstacles. As a result, once a path is generated, it cannot adapt to moving obstacles, making it inherently unsuitable for dynamic environments. Based on the observation of the success cases of the A* and RRT algorithm, we found that they depend on whether the person is on the pre-planned path. If they are, a collision will occur 100%; if not, the robot may succeed. Therefore, we enforced an encounter with a pedestrian in every trial, causing A* and RRT to fail 100% of the time. Consequently, we have not included them in the statistical analysis.

**Fig. 13:**
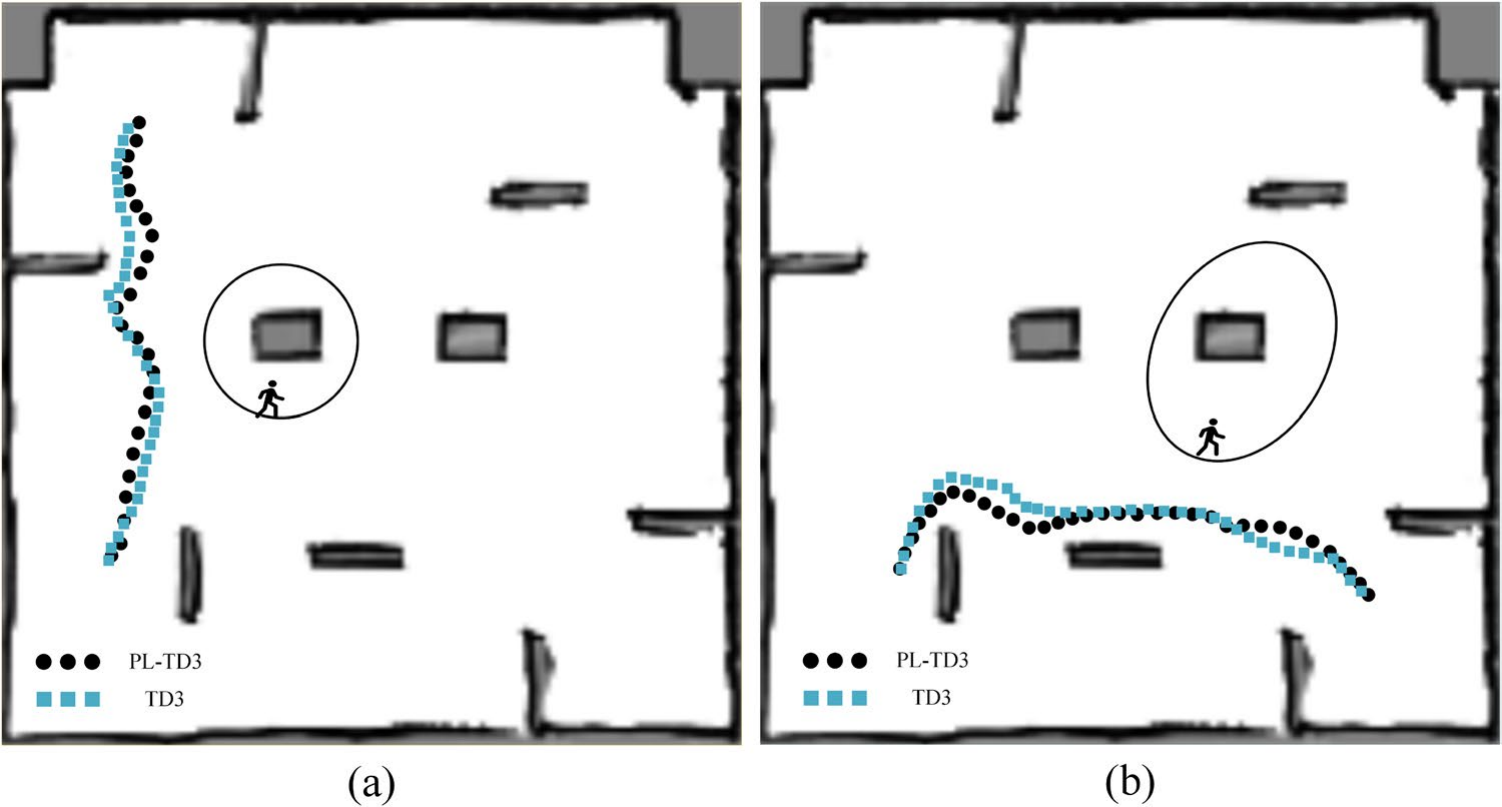
A set of sample path maps of the robot in a real-world environment, where circles denote pedestrian trajectories. (**a**) The robot encounters the human in a narrow area. (**b**) The robot encounters the human in a wide area.

The experimental results are shown in Table 4, and a set of sample paths planned by the PL-TD3 and TD3 algorithms are shown in Fig. 13 (circles denote pedestrian trajectories). The results indicate that PL-TD3 outperforms the other methods in both wide and narrow areas. Specifically, in the wide area, the success rate of PL-TD3 is 14% higher than TD3. In terms of path length, the route executed by PL-TD3 is 0.26m shorter than TD3.

The experimental environment demonstrates that the PL-TD3 algorithm exhibits strong adaptability to environmental changes, significantly outperforming the TD3 algorithm in terms of success rate and path length. video of real-world experiment

**Table 4:** Comparison of main evaluation indices for narrow and wide area scenarios in the real-world environment.

ENV	Algorithm Name	Main Evaluation Index			
		Average Path Length (m)$\downarrow$	Number of Successful Instances$\uparrow$	Success Rate (%)$\uparrow$	Collision Rate (%)$\downarrow$
ENV (Encounter in Wide Area)	PL-TD3	**6.02**	**87**	**87%**	**13%**
	TD3	6.28	73	73%	27%
ENV (Encounter in Narrow Area)	PL-TD3	**6.07**	**88**	**88%**	**12**%
	TD3	6.43	71	71%	29%

The bold type represents the algorithm having the best performance.

## Discussion

Our results indicate that the PER mechanism and the LSTM network play a critical role in mobile-robot path planning. Compared to existing approaches, PL-TD3 seamlessly integrates PER, LSTM-based temporal modeling, and the TD3 reinforcement learning framework to deliver consistently stable, high-performance navigation in dynamic environments. This study has two main limitations. First, neither our simulation nor real-world experiments incorporated sensor-error compensation, which may lead to significant performance degradation under high sensor noise. Second, the manually designed reward functions in PL-TD3 lack sufficient generalization, limiting its adaptability to new tasks. Future work should address these issues by developing effective sensor-error compensation strategies and designing more generalizable reward functions to further enhance the algorithm’s robustness and applicability.

## Conclusion

To address the sampling efficiency problems of the TD3 path planning algorithm and the challenges of using feedforward neural networks in dynamically changing environments, our work presents the PL-TD3 path planning algorithm for mobile robot. By incorporating PER strategy and LSTM neural network, the sampling efficiency of TD3 is enhanced, breaking the correlation between samples and improving its performance in long-term planning environments. This enhancement makes PL-TD3 more effective and robust in a wider range of application scenarios in dynamic environment. To verify the efficiency and stability of the algorithm, we conducted experiments in environments with static and dynamic obstacles, as well as tests for generalizability and practical effectiveness, comparing our approach with other similar path planning algorithms. In the narrow and wide area scenario testing experiments, PL-TD3 demonstrated a 13% reduction in execution path length, a 22% reduction in execution time, and a 20.3% increase in success rate compared to TD3. In the generalizability tests, PL-TD3 achieved a 12.5% reduction in path length, a 29.2% reduction in execution time, and a 26.6% increase in success rate compared with TD3.

Furthermore, we verified the practicality, effectiveness, and robustness of the proposed PL-TD3 algorithm in real-world scenarios within dynamic environments.

For future work, our research will focus on further optimizing the smoothness of path planning, with plans to introduce trajectory smoothing algorithms to generate more smooth planned trajectories. Additionally, in many real-world tasks, manually designed reward functions usually lack generalization capabilities. Therefore, we intend to employ Inverse Reinforcement Learning (IRL) techniques to automatically infer reward functions by analyzing expert demonstrations, thereby enhancing the adaptability and generalization of the algorithm.

## Data Availability

Data used in this article will be made available on request.
